# Exploring the use of smartphone monitoring for young adults with Tuberous Sclerosis Complex (TSC): a qualitative analysis

**DOI:** 10.1186/s13023-026-04287-5

**Published:** 2026-04-22

**Authors:** Kate Fifield, Katie Blackford, Benjamin Snaith, Anishka Singhania, Charlotte Tye, Sara Simblett

**Affiliations:** Department of Psychology, Institute of Psychiatry, Psychology & Neuroscience, King’s College London, London, UK

**Keywords:** Tuberous Sclerosis Complex, Ecological momentary assessment, Experience sample method, Focus group, Interview, Qualitative, Young adult, Smartphones, Rare disease

## Abstract

**Background:**

Smartphone ecological momentary assessment (Smart EMA) has the potential to address limitations in standardised cross-sectional self-report assessments. Young adults with rare genetic conditions, including Tuberous Sclerosis Complex (TSC), may further benefit from Smart EMA as it may help overcome associated cognitive difficulties in self-report and mental health stigma. Framework analysis was used to explore the hypothetical acceptability of the use of Smart EMA in monitoring the daily experiences of young adults with TSC.

**Results:**

14 young adults with TSC (Female = 9, age range 17:28) participated in online focus groups and interviews. Four main themes were generated. The first two themes describe the daily experiences of the participants (Understanding a New Young Adult Identity and Communicating Mental Health). The third theme explains how Smart EMA could be integrated into their everyday lives to support them (How Smart EMA Could Help). The final theme describes what would help or stop them from using the app (Hypothetical Barriers and Facilitators). This includes a need for personalisation, ease of use and unwanted unpredictability.

**Conclusions:**

There are multiple ways in which Smart EMA could be integrated into supporting young adults with TSC, both clinically and in research. However, consideration must be taken into the design and protocol of Smart EMA to ensure its appropriateness for the varied abilities of the young adult TSC population. Further testing of hypothesised moderators is required to conclude Smart EMA is feasible and acceptable for young adults with TSC and other rare genetic conditions.

**Supplementary Information:**

The online version contains supplementary material available at 10.1186/s13023-026-04287-5.

## Background

Tuberous Sclerosis Complex (TSC) is a rare genetic condition characterised by the growth of benign lesions in multiple organ systems, including the brain [1]. TSC is hugely variable in its manifestation ranging from premature death in children to adults who go undiagnosed for the majority of their life [2]. In addition to physical manifestations (such as epilepsy), most individuals with TSC will experience TSC-associated neuropsychiatric disorders (TAND), an umbrella term to describe the behavioural, academic, neuropsychological, psychiatric, intellectual and psychosocial problems in TSC [3]. This can include Autism, ADHD, Intellectual Disability, Anxiety, and Depression. The presence of TAND is associated with increased burden and reduced quality of life [4] and has been highlighted as an important area of research and improvement of care for families and individuals with TSC [5]. Another feature of TSC is the change in physical and TAND phenotypic presentations during development within individuals [6]. Cardiac and behavioural problems are prevalent in early childhood shifting to lung, kidney and psychiatric difficulties in early adulthood [7, 8]. Although symptoms may change, the interplay between physical and mental health in TSC is consistent throughout the lifetime [9, 10].

Young adulthood is a key transitional period, especially for those with long-term health conditions (LTC’s) [11]. Changes in clinical care, family support and legal processes in addition to typical development changes in education and work must be managed by young adults with LTC’s. However, little research has been conducted on mental health outcomes and their impact on social well-being and quality of life in young adults with TSC. Features of TAND, including social isolation and depression, have been shown in adults with TSC, but very few adults report follow-up of psychological concerns in adulthood [12].

The reasons for this may be due to a multitude of circumstances. Substandard healthcare transition due to inadequate guidance in self-managing their care needs and a loss of trusted support networks may result in young adults with chronic diseases disengaging from services [13, 14]. This can exacerbate mental health and other TAND difficulties already present from teen years [15]. For TSC individuals who have to coordinate their care across multiple health specialities, this can be especially difficult. Furthermore, the balance of striving for independence in young adulthood [16] coupled with the distrust in new adult services due to mismanagement of their transition, may act as a barrier to re-engaging with clinical services [17]. Young adults in the UK have also highlighted how stigmatising beliefs around mental health act as a barrier to seeking support [18]. This can also translate into low participation rate in health research [19, 20].

To address this, telehealth and smartphone health applications have been recommended to improve the care of individuals with TSC and support TSC research [21, 22]. In 2023, 91% of UK residents were smartphone users [23], increasing to 99% for young adults in the UK (aged 16–34) [24]. This may be especially beneficial for young adults with TSC as they are required to be more in control of their personal health and increasingly use their smartphones as sources of information, planning and management of their health [25]. Smartphones are also discreet and easily accessible which may overcome mental health stigma as a barrier to seeking support and increase involvement in mental health research.

The development of smartphone technology has allowed previous assessment techniques carried out on paper to be administered more easily and less burdensome via smartphone apps. One methodology is Ecological Momentary Assessment (EMA) which is a novel method of capturing everyday experiences or symptoms via self-report [26]. Using smartphones (Smart EMA), individuals receive repeated notifications (at least twice a day) for a select period to self-report their experiences, feelings, and thoughts ‘in the moment’, mainly via questionnaires but sometimes via multimedia. This methodology aims to overcome retrospective bias and collect data in real-time, real-world settings, foregoing the unnaturalistic, lack of context settings of lab-based data collection [26]. Smart EMA may be used in young adults with TSC to assess current TAND and other physical health symptoms (epilepsy seizures). It may help to consolidate TSC symptoms, both physical and mental, under one measurement tool, which can be used to unite an adult’s individual care where a previous paediatrician would have. This is currently being done using cross-sectional measures in the TANDem app aimed at reducing the identification and treatment gap for TAND [27]. Smart EMA may supplement this by providing increased highly detailed individual daily life observations to inform treatment and management. Additionally, Smart EMA can be combined with passive physiological monitoring using wearables (e.g. Smartwatches), increasing our understanding of complex psychological and physiological processes [28]. This may be especially useful for young adults with TSC who can present with varying unique profiles. Furthermore, for individuals with TSC who may have difficulty recalling long-term experiences, i.e. those with cognitive impairment, which is highly present in the TSC population, Smart EMA can overcome these difficulties by limiting recall to just a few hours. Finally, it may also lead to in-the-moment intervention which evidence is emerging in psychiatric disorders [29].

Since the first smartphone EMA study was published in 2010 [30], there is growing evidence that Smart EMA is feasible in populations of adults with mild to moderate intellectual disability (ID) [31], mental health difficulties [32], neurodevelopmental disorders [33], physical health conditions [34] and young adults [35]. However, this study builds on a previous systematic review of Smart EMA in neurological, neurodevelopmental and neurogenetic conditions which found a lack of focus on maximising EMA feasibility in these clinical populations [36].

A critical step in using Smart EMA is to establish how to engage people in this type of novel data collection. Previous qualitative research has highlighted the role of several factors, including the health status of the person at the time of data collection, how usable, convenient, and accessible the device or process is, the perceived utility of their efforts and, relatedly, general motivation [37]. However, there has been no research into how individuals with TSC may engage with Smart EMA. To ensure that these factors are taken into consideration when using Smart EMA methods with young adults who have TSC, it is important to explore how young adults with TSC make sense of EMA and how it can be integrated into their daily experiences and involve them in the design process. Furthermore, it was felt important to explore these views at the initial stages of the Smart EMA feasibility and acceptability research so the young adults’ views can be included when choosing a suitable EMA app.

## Method

### Aims

This study aims to: explore the attitudes and opinions of young adults with TSC regarding the hypothetical feasibility of using Smart EMA in their daily life; understand the accessibility and appropriateness of EMA for young adults with TSC which will guide the protocol development for a subsequent in vivo feasibility study; and gather information on young adults with TSC’s technology and smartphone use.

### Design

The design is a mixed-method study based on focus groups (FG), semi-structured interviews (INT) and questionnaires. As this was the first time Smart EMA was being explored in TSC, we wanted to elicit open discussions about the opinions and views that young adults with TSC may have about Smart EMA which qualitative methods can facilitate [28, 38]. Focus groups have been used previously to gather participant’s views and priorities on m-health technology [37, 39] and EMA Focus groups have been used [40]. Interviews were also offered to increase recruitment as individuals with TSC are more likely to experience social, intellectual or communication difficulties that may prevent them from accessing focus groups.

### Ethics

Ethical approval was granted by King College London Health Faculties Research Ethics Subcommittee (Ref: HR/DP-22/23-34436). Written informed consent was taken for all participants.

### Participant selection and sampling

Using convenience sampling, participants were recruited through the prospective longitudinal Tuberous Sclerosis 2000 (TS 2000) study cohort [6], the Tuberous Sclerosis Association (TSA) Charity research network and their respective social media. Participants were eligible if they were a young adult (aged 17–30 years old) with a diagnosis of TSC. All participants had to have the capacity to consent, and this was measured using a capacity checklist at screening. See Table 1 for description of participant characteristics.

**Table 1:** TYP participant characteristics

		TYP (n = 14)	TYP FG (n = 9)	TYP interview (n = 5)
Age (years), mean (range)		21.93 (17:28)	22.11 (17:28)	21.6 (18:25)
Gender, % (N)	Female	64.3 (9)	66.7 (6)	60 (3)
	Male	28.6 (4)	22.22 (2)	40 (2)
	Non-binary	7.1 (1)	11.1 (1)	
Ethnicity, % (N)	Asian/Asian British - Indian	7.1 (1)	11.1 (1)	
	White - British	85.7 (12)	77.8 (7)	100 (5)
	White - Irish	7.1 (1)	11.1 (1)	
Employment, % (N)	Employed full time	21.4 (3)	22.2 (2)	20 (1)
	Employed part time	14.3 (2)	11.1 (1)	20 (1)
	Student	42.9 (6)	33.3 (3)	60 (3)
	Unemployed - looking for work	21.4 (3)	33.3 (3)	
Education, % (N)	Higher or secondary or further education	57.1 (8)	55.6 (5)	60 (3)
	Secondary school up to 16 years	14.3 (2)	11.1 (1)	20 (1)
	University	21.4 (3)	22.2 (2)	20 (1)
	Other*	7.1 (1)	11.1 (1)	
Age (months) at diagnosis, mean (range)		11.6 (0:36)	6.8 (0:18)	11.8 (5:36)
TSC genotype, % (N)	TSC1	21.4 (3)	22.2 (2)	20 (1)
	TSC 2	21.4 (3)	11.1 (1)	40 (2)
	Not tested	7.1 (1)		20 (1)
	Unknown	50 (7)	66/7 (6)	20 (1)
TSC Symptoms, % (N)	Brain	78.6 (11)	66.7 (6)	100 (5)
	Nervous System	85.7 (12)	88.9 (8)	80 (4)
	TAND	78.6 (11)	77.8 (7)	80 (4)
	Kidney/Urinary Tract	64.3 (9)	66.7 (6)	60 (3)
	Heart	57.1 (8)	55.6 (5)	60 (3)
	Skin	85.7 (12)	88.9 (8)	80 (4)
	Lung	35.7 (5)	22.2 (2)	60 (3)
	Other**	7.1 (1)		20 (1)

* Level 4 certificate with Ulster university **Low muscle tone

### Researcher characteristics

Interviews were conducted by only KF (Female PhD student working on the research project). Focus groups were conducted by KF and supported by two female undergraduate placement students (KB & AS) and one male undergraduate final-year student (BS). KF met with the student immediately after each focus group to debrief and make notes. KF had previous experience running research interviews in a clinical setting and running focus groups in a university student sample. KF also had clinical therapeutic experience of working with similar participants. These characteristics were acknowledged to have influenced the final interpretation. Please see Additional file 1 for a reflexivity statement. Participants had no prior relationship to the researchers or knowledge of researcher’s motivations. However, the majority of participants had been recruited from a longitudinal cohort study [6], which may have influenced their views on the proposed research tool.

### Setting

The whole study procedure was conducted via the telephone and online. All interviews and focus groups were conducted using Microsoft Teams. All questionnaires and consent forms were completed via Qualtrics [41].

### Measures

The Modified Computer Self-Efficacy [42] was used to assess hypothetical self-efficacy with using Smart EMA. This scale has been validated in individuals with disabilities. Minor modifications were made to the wording of the scale (‘Unfamiliar technology’ was changed to ‘Smartphone app’). Total scores are summed out of 100 with higher scores indicating greater technology efficacy. All other measures were bespoke.

### Study procedure

All participants were screened for their eligibility over the phone. They then completed consent forms via Qualtrics. The study consisted of two phases. Phase 1 involved the TSC Young Person (TYP) completing an online questionnaire asking about their smartphone use (See Additional file 2). They also completed a demographics questionnaire. In Phase 2, participants were asked if they wanted to take part in a focus group, an individual interview or an interview with a chosen Key Support Person (KSP). KSP’s were defined as parents, carers (informal and formal), partners or other family members. If participants indicated they wanted to take part in an individual interview with a KSP, the KSP was contacted and consented as a ‘KSP non-participant.’ The KSP non-participants were there to only offer support to the TYP and were not asked about their opinions or experiences. The focus groups and interviews were semi-structured using a topic guide (available on request) and adapted from a previous qualitative study [37]. Word clouds were used as a ‘icebreaker’ before the main discussion began (see Additional file 3). During the groups/interviews, a PowerPoint with examples of EMA apps were shown, including how and what type of questions might be asked and an example of Smart EMA structure. These were guided by a systematic review of Smart EMA in similar clinical populations [36].

At the end of the focus groups or interviews, all participants completed the Modified Computer Self-Efficacy Scale.

### Analysis

Focus groups and interviews were audio and video recorded, transcribed verbatim and checked. All transcripts were anonymised. Qualitative analysis was conducted using NVivo (version 14; QSR international, Melbourne, Australia). Framework analysis [43] was applied as we had predefined areas we wanted to understand (facilitators and barriers to Smart EMA) but were also open to emergent codes and themes within the context of the participants experience of living with TSC. After familiarisation, KF developed an initial basic codebook informed by predefined questions and emergent ideas from the familiarisation stage. KB then used this codebook to recode the data. KF and KB then worked together through many iterations to develop a theoretical framework or ‘codebook’. This framework was applied to the transcripts using a framework matrix to understand the ‘fit’ of the data and quotes were assigned to the relevant categories. Participant’s quotes were then summarised and overall summaries for each category and case were defined. Patterns and concepts were then sought and labelled as themes and sub-themes.

After the first refinement of themes and subthemes, these were shared with the participants. Four participants responded and one code was changed due to not fitting with their experience: “Using the app increases self-confidence”. After re-coding, all four participants agreed that the rest of the results matched their experience.

Results were reported following The Consolidated Criteria for Reporting Qualitative Research (COREQ checklist) [44], see Additional file 4 for checklist.

Descriptive and computer self-efficacy scale statistics were analysed using SPSS statical analysis software (Version 29, IBM). Participant demographics were compared between interview type (FG or INT) to check for sample variation and were also compared to the TS 2000 cohort to check for sample bias. The TS 2000 study is a UK-representative prospective longitudinal study of the natural history of TSC. Newly diagnosed TSC cases aged between 0 and 16 years and born between 2001 and 2006 were recruited into the study and have been followed at regular intervals [6].

For the Modified Computer Self-Efficacy scale, univariate analysis comparing participant characteristics and total scores were conducted. Non-parametric tests were used due to the confidence scale total score violating the assumption of normal distribution. Means comparisons were tested via Mann-Whitney U test and Kruskal-Wallis H test for categorical variables. Bivariate analysis using Kendall’s’ Tau-b test was used for continuous variables.

## Results

### Participant characteristics

**Fig. 1:**
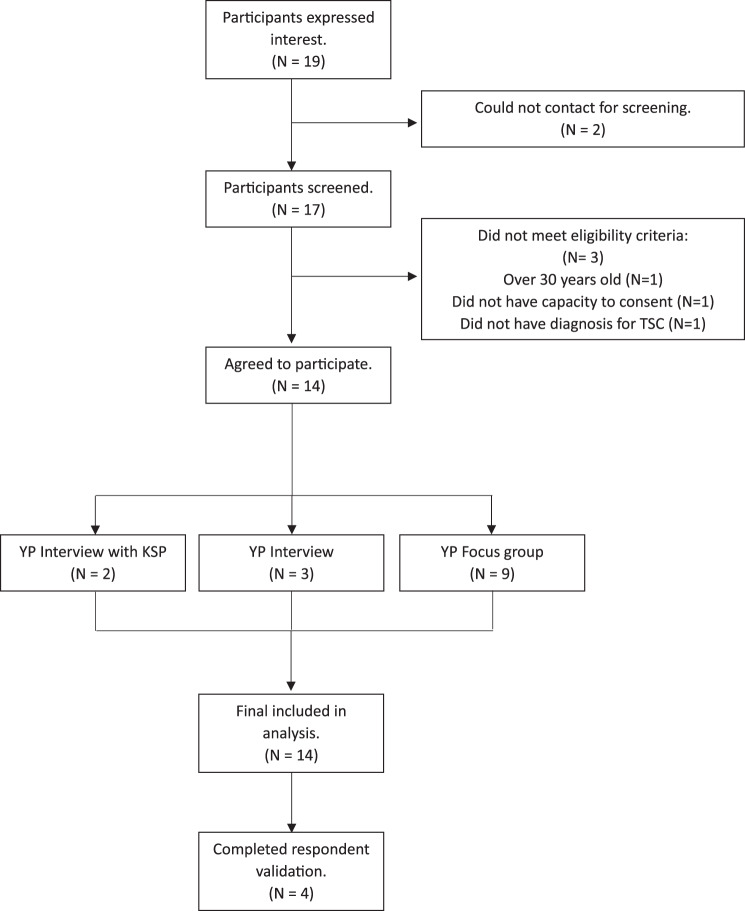
TYP recruitment flowchart

14 participants were recruited. Two focus groups were conducted with 9 TYP (group 1: *n* = 5, group 2: *n* = 4). Both groups lasted 1 hour. Five interviews were conducted with TYP, with 2 young adults having a KSP present. The mean interview time was 52 minutes. The reasons for the interview choice included: social and communication difficulties (2), social difficulties (1), other – easier for schedule (1) and one TYP could not attend either focus group so was interviewed instead. See Figure 1 for recruitment flowchart. See Table 1 for participant characteristics and Table 2 for smartphone use.

**Table 2:** TYP smartphone use

		TYP (n = 14)	TYP FG (n = 9)	TYP interview (n = 5)
Smartphone use, % (N)	Mobile	100 (14)	100 (9)	100 (5)
	Smartphone	100 (14)	100 (9)	100 (5)
Years using Smartphone, mean (range)		9.5 (4:14)	9.22 (4:14)	10.33 (7:12)**
Use, % (N)	Phone calls	92.9 (13)	100 (9)	100 (4)*
	Text messaging	85.7 (12)	100 (9)	75 (3)*
	Shopping	57.1 (8)	77.8 (7)	25 (1)*
	Banking	78.6 (11)	77.8 (7)	100 (4)*
	Emailing	78.6 (11)	77.8 (7)	100 (4)*
	Exercising	21.4 (3)	22.2 (2)	25 (1)*
	Social networking	92.9 (13)	100 (9)	100 (4)*
	Navigating with maps	71.4 (10)	88.9 (8)	50 (2)*
	Entertainment	78.6 (11)	100 (9)	50 (2)*
	Other (studying)	7.1 (1)	11.1 (1)	
Tracking health apps, % (N)	Yes	28.6 (4)	33.3 (3)	25 (1)*
Other devices, N	Computer	4	2	2
	Laptop	8	4	4
	Tablet	3	3	
	Game console	1	1	
Tracking health apps on other devices, N	No	100 (14)	100 (9)	100 (5)
Easy to use, % (N)	Extremely easy	64.3 (9)	66.7 (6)	60 (3)
	Somewhat easy	35.7 (5)	33.3 (3)	40 (2)
Wearables, % (N)	Yes	35.7 (5)	44.4 (4)	20 (1)
	No	64.3 (9)	55.6 (5)	80 (4)

*Missing 1 participant’s data

**Missing 2 participants data

### Comparison between FG and interview and TS 2000 cohort

There were no significant differences between those who participated in the FG or INT (*p* > 0.05). There were also no significant differences in age, gender, age at diagnosis and TSC genotype between the TYP, the TYP who requested a KSP in this study and the TS 2000 cohort (*p* > 0.05).

### Smart EMA self-efficacy

The average app self-efficacy score was *M* = 85.86 (SD = 13.36). For the TYP, ‘if had used similar apps before’ was rated with the highest confidence. ‘If there was no one around to tell me what to do’ was rated with the lowest confidence. See Table 3 for scores.

**Table 3:** TYP responses to the modified computer self-efficacy scale after the FG/INT

Item	Score										
	1	2	3	4	5	6	7	8	9	10	Total
Time to complete	0	0	0	0	0	1	1	0	4	8	129
Help started	0	0	0	0	1	0	0	2	3	8	128
Demonstration	1	0	0	0	0	0	1	0	2	10	126
Help available	0	0	0	0	1	0	2	0	3	8	126
Observed use	0	0	0	0	1	1	0	1	5	6	124
Similarity to other apps*	0	0	0	0	0	0	1	3	1	8	120
Only built in help	1	0	0	0	0	0	3	1	3	6	117
No help available	0	0	0	0	1	0	4	2	3	4	116
Only manual instructions	0	0	0	0	2	0	1	3	2	5	109
No previous use	0	0	0	0	2	2	3	3	0	4	107

(Scored from 1 = not at all confident to 10 = completely confident)

*Missing one response

**Fig. 2:**
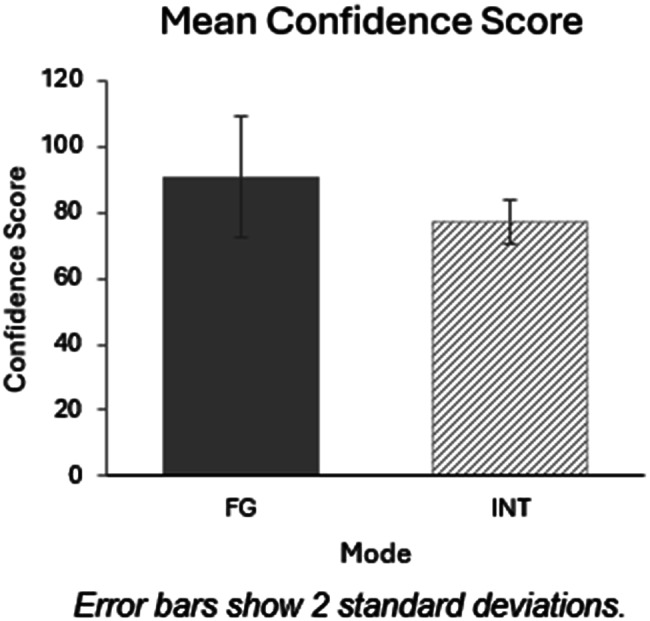
Bar chart showing TYP mean confidence scores for FG/INT

TYP who took part in a focus group reported having greater confidence in their ability to use Smart EMA (*M* = 90.78, SD = 6.67) compared to those who took part in an interview (*M* = 77, SD = 18.4) and this was significant (*U* = 7.5, *p* = 0.045). See Fig. 2. There were no other significant differences between participant characteristics and confidence scores (*p* > 0.05).

## Qualitative results

**Fig. 3:**
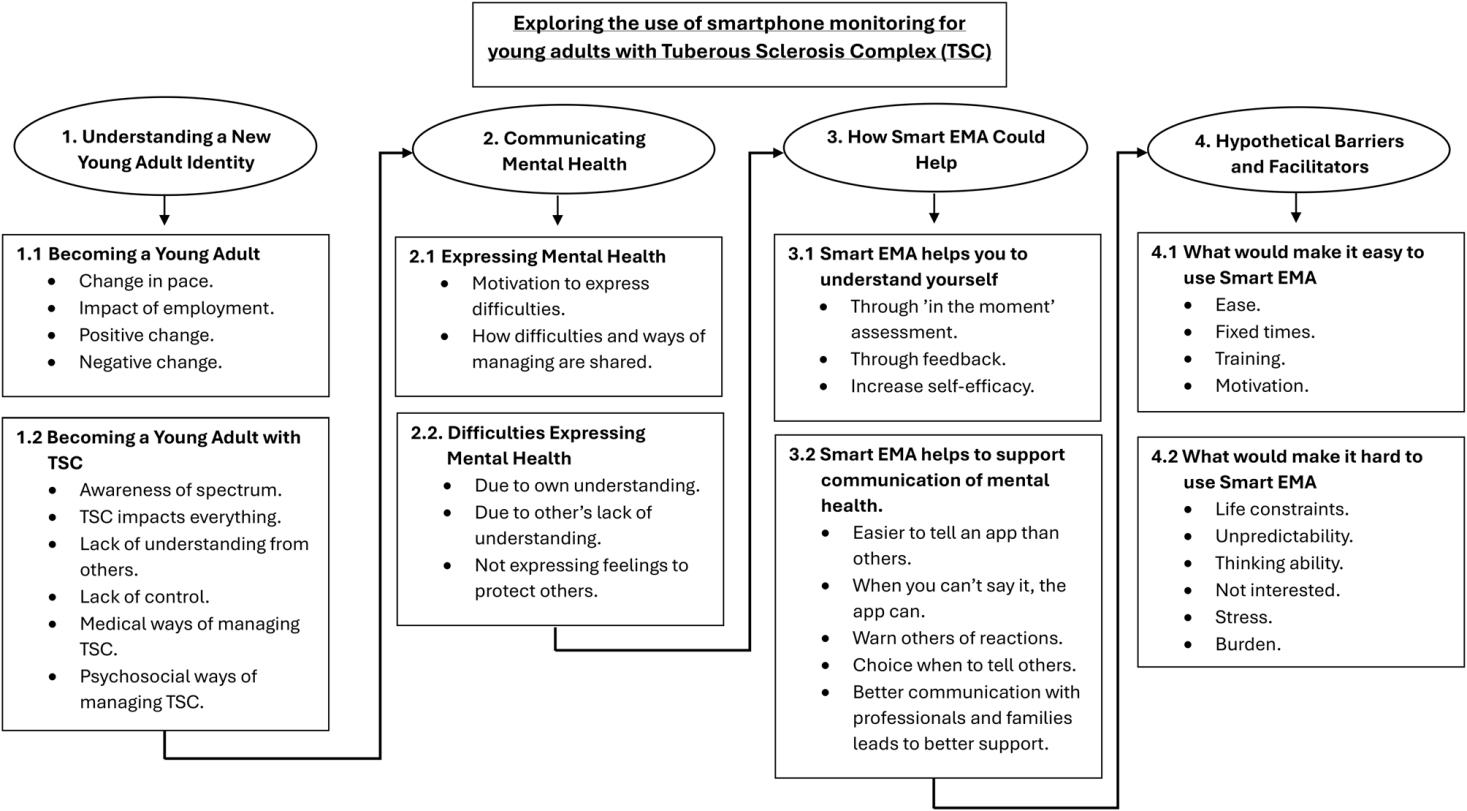
Main themes and sub-themes

The young people’s views were captured in four major themes with sub-themes divided across them (See Fig. 3). The first two themes described the general experiences of the young adults with TSC, with the third theme describing how Smart EMA could support them in these daily experiences and difficulties. The fourth theme describes the hypothetical barriers and facilitators of Smart EMA.

### Understanding a new young adult identity

This theme describes the participant’s general experiences of becoming young adults but also how they specifically mention the challenges of becoming a young adult with TSC.

#### Becoming a young adult

Participants spoke about a change in pace in their lives. Some participants described how they had increased responsibility such as paying their own bills and having a job which led to a feeling of ‘busyness’. Other participants spoke about they are finding it “slow” and boring, often related to not seeing their friends as much.

Participants also spoke about the impact of employment on their wellbeing. Some talked about how having a job can improve their confidence and can create stability in a young adult’s life. However, they also spoke about how employment can affect their mental health and overall well-being, often in a negative way.

*PID 1: “I mean the thing is when I left that job, which was because of my mental health like and then when I got to this job, I guess maybe it is also partly about like the people you work with and like when I settle down into this job. I guess things sort of started to change a bit for my mental health.”*

Some participants spoke about the positive changes that have occurred since becoming a young adult. They like being treated like an adult and having increased independence so find it easier being a young adult than being a teenager. However, some participants commented that they have lost friendships since becoming a young adult. One participant spoke about how they could no longer play tennis with their friends as they did as a teenager, which was important to them.

*PID 20: “I feel like people treat me more like an adult now which I like, I guess, I’m not kind of getting … I dunno people-people talking to me as if I was a child and I didn’t really like that to be honest but, there’s less of that now which I like.”*

#### Becoming a young adult with TSC

Participants were aware that TSC is a spectrum disease and that not all individuals with TSC will have the same experiences. They were also aware that those with other conditions, such as epilepsy or Autism, also have different challenges. Participants talked about how having a spectrum condition sometimes made it difficult to find people to share their experience with as no one had the same journey with TSC as they had had.

*PID 1: “I think the person in the welfare department was like, OK, what probably makes it hard for you is that … you are actually quite able sometimes to say to your so called neurotypical friends exactly the things the things their other neurotypical friends might say so what makes it quite hard for you is that you sort of in the middle in effect where you are not quite neurotypical but you are not disabled or classically autistic enough to quite fit in with other autistic people.”*

Participants spoke about how TSC impacts every part of their life. This included their thinking ability and processing and understanding others. Another area that TSC affected was their mental health such as causing anxiety and depression. TSC also impacted their physical health including the impacts of epilepsy or problems with their kidneys.

*PID 3: “TS can like … you can be going like with a good pace within your life you could be enjoying it … and then something can could come up with your mental health or physical health due to TS and then it could like it could throw you off the boat so to say so you’d be riding smoothly in a boat and then all of a sudden like you could have a bad couple of weeks or a bad day with your mental health or with TS in general with your general health with your kidneys or with … or just with your processing and understanding people around ya so can be quite uh … like PID 10 says lonely and a bit confusing and unpredictable it’s can be quite difficult.”*

Because TSC impacted so much of their lives, the young adults described having feelings of being out of control. This exacerbated the mental health difficulties they already faced.

However, participants spoke about ways they tried to manage these feelings. One way was to create as much control as possible in their lives, such as keeping the same routines and planning events and activities with their friends. They also spoke about other psychosocial ways they managed their TSC which included physical activity, creative activities and gaming.

*PID 2: “I, I play The Sims because it allows me to have control over someone’s life if I can’t have control over mine.”*

As part of managing the impact of TSC, participants also spoke about the medications they take, whether this was epilepsy medication or anxiety medication. However, this had negative side effects. These included how it impacted their memory and caused “brain fog” and tiredness. These side effects also lead to difficulties with their mood and well-being.

Even though participants had ways to manage their TSC, they still expressed how others did not understand how TSC impacts their daily lives. They spoke about how they felt disadvantaged at work and that employers didn’t know how to support them.

*PID 19: “Yeah, I mean I guess like appointments have always been a struggle … managers have sort of, uhm, not managed me too well or then like made me redundant so soon cause they didn’t know how to manage my conditions”.*

They also spoke about the lack of research around TSC and where they can find help for their difficulties. This fed back into the difficulties of TSC being a spectrum disease and that often information they saw about TSC was not relevant to them.

They also spoke about how having TSC impacts their social relationships. For example, how friends do not understand what TSC is, how it affects them or how young adults find it hard to process others. One participant mentioned how her friends joke about her memory difficulties *“my friends make a joke of it they always say like if I had a pound for every time you said oh what was that or like do-do you not remember um I would be a millionaire”.* This led to feelings of loneliness and feelings of being misunderstood even by their friends.

### Communicating mental health

This theme describes how the participants want and need to talk about their feelings and emotions. It explains how the young adults currently share their difficulties and their ways of managing. However, there are still barriers and challenges to participants talking about their feelings and emotions.

#### Expressing mental health

Participants spoke about why they currently talk about how they are feeling. One participant talked about how it is normal to express feelings because we are human, that’s what we are meant to do and should be allowed.

*PID 19: “I want to show my emotions cause I’m just human”*

The participants also spoke about how because their mental health had the biggest impact on them, they had to share this, otherwise, it would get too much and become overwhelming. This would also lead to increased stress and bring on other TSC physical symptoms.

*PID 19: “cause sometimes when I can’t cope, I either go into breakdown and start crying excessively or I get angry or physical symptoms will come on if I’m not coping with the emotions.”*

Participants then spoke about how they currently talk about their feelings and how they manage these. The most popular ways were talking with their parents and their friends, along with talking to professionals such as a therapist. Other ways also included sharing with the TSC community and having peer support.

*PID 6: (talking about their mother) “She’s generally the person I go to for things like this, because she understands because she has a very very very very minor case of TS but I got, the much bigger, much bigger case. But because she’s a nurse if I ever have anything like physical or mental going on then I go to her”*

#### Difficulties expressing mental health

Participants spoke about how sometimes they didn’t even know what they were feeling or why they were feeling certain emotions. This made it difficult to tell others and ask for support. Another barrier to seeking support was that participants either did not have a good family support network or were unsure of the reaction they would get if they shared with their family. Some participants spoke about how they wouldn’t share their feelings with others, especially their families, as they didn’t want to worry them or cause them stress.

*PID 3: “I generally try and have her* (mum) *not worry as much as I can because she’s worried up to this point in my life anyway she’s worried for too long already, so …”*

Some participants also spoke about how it was difficult to explain their feelings to health professionals as appointments could be overwhelming and difficult to manage. This was either due to memory difficulties, or issues with understanding their questions or communicating their experiences.

*PID 7: “Because it’s not that you don’t like them, but it’s just because sometimes their questions like you zone in and out or the medication and other reasons”.*

### How smart EMA could help

This theme describes how the young adults understood Smart EMA and how it could be integrated into their daily experiences. Following on from the two previous themes, two concepts were discussed; (1) how Smart EMA could help individuals understand why they are feeling certain ways and (2) how Smart EMA could help individuals share their mental health difficulties with other people to access support.

#### Smart EMA helps you to understand yourself

Participants spoke about how they thought being able to answer simple, individual questions ‘in the moment’ would help them have more accurate responses. This was due to the simple questions making it easier to visualise their feelings or helping to overcome learning difficulties.

*PID 17: “I think sometimes it’s hard to kind of like put into words as well. So if you’ve kind of got like a prompt there that kind of like gives examples, kinda like “okay, you know what, that is—that’s kind of like how I feel” and then it’s easier to answer.”*

Being able to see previous answers was interesting to the participants. They wanted to see the ‘full picture’ and patterns or trends. This would help participants understand their own strengths and weaknesses and the relationships between their feelings, behaviours and what they were doing in those moments.

*PID 21 “They are relevant because it kind of understands us. It’s like we are being understood. Because we, uh—that moment, we ourselves will understand that something is going to happen or something like that. So it kind of makes us feel that—“okay, we are feeling a little down” we should like sit down or rest a bit, rather than going for another task.”*

This would in turn help the participants take personal action such as trying to improve their reactions to situations or manage their feelings. This would help to reduce their reliance on professional support for their emotional well-being.

*PID 19: “but maybe using app would help as well for using at home so maybe I won’t have to rely so much on a counsellor so often.”*

#### Smart EMA helps to support communication of mental health

Some participants felt they would feel more comfortable telling their feelings to Smart EMA rather than straight to others, including professionals and families. This would be helpful when participants cannot tell their feelings to others, either due to not feeling comfortable or due to illness. By using Smart EMA, participants can also choose when they want to share these feelings with others or keep them to themselves.

*PID 2: “It comes back to the whole struggling with the memory thing, it means that I don’t have to remember how I’ve been feeling I can just get it up on my phone and tell the doctor.”*

By using Smart EMA, participants could also tell others what annoys them or makes them feel sad. This would then help others not to do these things and prevent the individual with TSC from reacting in a negative way.

*PID 14: “Or like you know something happens you know what I mean like something really negative happens and I might like, uh see what the situation is, at least I know like I can warn them in advance.”*

Participants spoke about how if they could use Smart EMA to tell professionals and families what they were feeling, this would lead to better understanding. Smart EMA would help them remember everything and explain it in an easy way. This would then lead to professionals and families being able to offer better support.

*PID 21: “Uh, you could show it to your partners or families, they could—maybe when they see it and they understand that you are actually going through something or your emotions are not right in that moment, they kind of note it and they kind of make—help you, the next time. So when you’re feeling scared or something, they make you feel better.”*

### Hypothetical barriers and facilitators

This theme describes what participants thought would make it easier to use Smart EMA and what would make it harder to use Smart EMA.

#### What would make it easy to use smart EMA

Participants spoke about how a Smart EMA app needed to be easy to download, understand and use. They also wanted the questionnaires to come at the same time every day and for them to know when the questionnaires were coming. They also spoke about how training would be important so they would know how to use a Smart EMA app.

*PID1: “So I think for me it would be like, better in the kind of job I’m in, if like you like see when the notifications are going to come.”*

Motivation for using Smart EMA was important for the young adults. They spoke about how they would be motivated to use Smart EMA due to receiving rewards, such as ‘well done’ messages or incentives. If Smart EMA was fun for example including the ‘brain games’, this would also motivate them to use a Smart EMA app.

*PID 19: “Uh, I’d probably happy completing the brain games by myself because they’re interactive and a bit of fun at the end of the day and they could be a challenge as well.”*

Participants also wanted to use the app to help support research. They understood that having a rare disease makes it important for them to take part in research.

*PID 1: “Um, I-I mean the thing for me is like … I-I-I guess I have been part of the TS study for like, well not anymore, but I was for quite a while and, uh anything like, like that like comes up about trying to improve the lives of, people with TS I-I uh sort of see as something, well good to get involved in so I guess I already have some sort of motivation to use the app. Uh-and-it’s about sort of trying like to find a way, well its about seeing what might be right for an adult with TS but its also for me about like trying to help people with TS um because its such a rare disorder that there’s just not much research on it.”*

#### What would make it hard to use smart EMA

Participants spoke about how because they are not allowed to use their phones at work or college, they would find it hard to answer lots of questionnaires a day. Also, because they didn’t know what their daily schedule would look like, it would be difficult to answer the questionnaires if they didn’t know what time they would appear. These factors meant that they might miss the questionnaires which they did not want to do.

Due to learning difficulties or cognitive difficulties, participants wanted a Smart EMA app to be simple with a mixture of pictures and words. Having a short time to start the questionnaires and not knowing when the questionnaires would come would be stressful for the participants as they would be worried if they missed answering the questionnaires.

*PID 3: “I think um the 15 minute like sort of lock time scares me a little bit cos its like oh I have to get it done and if you don’t get it done then that’s like one part of it not completed. Well yeh that scares me a wee bit.”*

Participants spoke about how if they were feeling ok, they would not want to be asked lots of questions as it would be unnecessary. They also wouldn’t want it to take a lot of time to complete. Also, if the app was “*boring”*, participants wouldn’t use it.

*PID 2: “Uh, yeh I’d be fine with it as along as it isn’t … gonna take me like half an hour everyday …”*

## Discussion

The present study explores young adults with TSC’s technology and smartphone use and their attitudes and opinions on the hypothetical feasibility of using Smart EMA in their daily lives. This study contributes to the evidence that Smart EMA could be useful and feasible in young adults with rare genetic diseases and other LTC’s, providing certain recommendations are accounted for.

### Technology and smartphone use

In our cohort, all participants used smartphones, mainly for phone calls and social networking, and just over a quarter used health apps at the time of the study. This quantitative data indicates that young adults with TSC who can participate in an online interview setting may have the resources to download and use an EMA app on their smartphone.

### How Smart EMA could help understand a new young adult identity

All participants described changes in their lives since becoming young adults. Interestingly, they spoke about this in relation to becoming a young adult with a chronic condition but also more universal changes which many young adults without chronic conditions experience i.e. employment and losing friendships [45]. The participants spoke about how employment can contribute to their wellbeing, both positively and negatively, supporting the widespread agreement of the value of good jobs for individuals [46]. The change in autonomy however was more felt through their experience of living with TSC. Managing the spectrum of TSC symptoms, as well as the side effects of medication, the young adults emphasised a need for control, which may not have been as obvious as a child when parents are responsible for creating that ‘control’ [47–49]. Interestingly, the participants didn’t specifically mention the loss of a parent role contributing to this lack of control but rather spoke about more general feelings of loneliness. This may indicate a difference between having a parent help with physical health needs compared to TAND symptoms which were more prevalent in the young adult’s discussion. This may also relate to the young adult’s conversation around ‘protecting’ their parents from feeling stressed about the young adult’s mental health difficulties, which has been reported in another rare genetic disease - Neurofibromatosis (NF1) [50]. This could be a strong motivator for young adults with TSC to use independent means of managing their TAND symptoms and in our groups, Smart EMA was seen as a way of potentially achieving this. In their review, Low and Manias [51] found limited effectiveness in improving self-management in adolescents with chronic conditions during transition to adult care. However, they only focused on mobile/web-based health interventions and not general monitoring which our adults predicted could be enough to promote self-intervention by themselves. Alongside personal reward for using Smart EMA, this cohort had a strong motivation for EMA contributing to rare disease research. This has been highlighted in other similar inherited cohorts using mobile health (mhealth) technology; Fabry disease [52], Tay-Sachs (TSD) and Sandhoff (SD) diseases [53] and provides a strong backing to future EMA studies in rare genetic diseases.

There is ample evidence indicating that young adults with chronic conditions learn ways to self-manage their symptoms and feelings [54, 55] which the young adults in this study also demonstrated. Staying active was a shared idea with the young adults and contributes to physical activity being a key concept in young people with a chronic genetic condition [56]. But, having to self-manage their TSC may have highlighted to the young adults the impact that the lack of understanding from others has on their day-to-day experiences. Employers were a key group identified to struggle with managing the young adults with TSC. This corroborates research from Both et al. [5] who stated that young adults with TSC wanted employer support during transition. Several other studies looking at employment in young adults with chronic conditions have also shown its impact on young adults reaching a feeling of acceptance with an adult identity [57, 58]. Smart EMA may be used to understand the impact of employment on young adults with health conditions and how to support positive employment experiences.

### How Smart EMA could help communicating mental health

Although the participants had found ways of self-managing their TSC, their mental health still played a large factor in their well-being. This supports the high levels of psychiatric disorders in adults with TSC and their impact on quality of life [12]. The cyclical nature of the participant’s mental health intertwined with their physical health symptoms is representative of other young adults with chronic health conditions [59]. In our research, the participants spoke of how Smart EMA could help them share their mental health difficulties with others. Lynch Milder et al. [60] found that satisfaction with social support mediated the effect on young adults with chronic health conditions social network size and physical functioning. Combined with our data, this could suggest that sharing mental health difficulties can interrupt the negative cycle of overwhelming feelings and TSC symptoms. However, in their seven-day EMA study, De la Barrera et al. [61] found that using social networks to share emotional feelings was associated with higher levels of depressive symptoms in young adults. Research into social relationships among young adults with chronic conditions also identified similar themes around how friends failing to understand the young person’s condition can be detrimental to their emotional well-being [62]. This discord highlights two things: Firstly, the type of data collection may impact our understanding of the benefits of socially sharing feelings. Cross-sectional questionnaires may not be picking up on the fluctuations of the real-time effect of sharing emotions with others and its impact on current mood. Secondly, socially sharing emotions may be useful but only when done in the right way. EMA could be used to determine the most beneficial way (which may be personal to each individual) or facilitate the sharing of information itself.

One barrier to communicating mental health was the young adults’ own understanding of their emotions. A potential mechanism of this could be alexithymia (recognising own emotions) [63]. There is consistent evidence that young adults with autism struggle with alexithymia [64] but, it has also been shown in patients with, chronic skin conditions [65] and neuromuscular disease [66]. Gerber et al. [67] quantified using EMA, that alexithymia symptoms, not autism, were associated with fewer social interactions. Gerber only measured one week of social interactions however, within our groups, participants who did not report a diagnosis of autism also shared alexithymia difficulties. This highlights the importance of helping young adults, with and without autism, recognise and understand their own emotions, to have beneficial social interactions. In a study conducted by Saikkonen et al. [68], an association in young adults between psychological distress, social support, and alexithymia was found. This contributed to their hypothesis that alexithymia prevents young adults from building stable social networks and in turn, seeking support from these networks for their emotional difficulties. Our data qualitatively supports this hypothesis as participants discussed how answering ‘in the moment’ simple questions would help them understand their emotions better and in turn, share them with others. This has also been reported by individuals with ID on their experiences of using EMA [69].

On the other hand, participants expressed that other people’s lack of understanding was also a major barrier to communicating their mental health difficulties. Our participants’ experience with health professionals mirrors work done by Kerin et al. [70] in 22q11 deletion syndrome. Their participants explained how their difficulties with communication and memory prevented conducive health appointments. This was moderated if the clinician was knowledgeable about their disease or listened to the young people when they explained they needed further support (i.e. writing down what was said in their appointments). This highlights that there is a need for better ways of sharing information between patients and clinicians in rare genetic disorders, and Smart EMA could be utilised to help bridge this gap.

### How Smart EMA could help in research

Understanding the feasibility and acceptability of Smart EMA in young adults with rare diseases is crucial. Better patient-reported outcomes are needed for both rare disease research and clinical support [71]. Smart EMA has been highlighted as a potential solution to the frequent failures of mental health clinical trials as assay sensitivity (can symptom measurement assess differences between treatment groups) cannot be confirmed [72]. Although general mobile health and remote assessment may help with sensitivity detection, the high frequency and real-time assessment nature of Smart EMA has already been shown to detect differences quicker than standard assessment in depression research [73]. It is currently being evaluated in The IMMERSE study, which is a multi-centre randomised controlled trial analysing the implementation of digital EMA into mental health clinical care across Europe [74].

Smart EMA can also overcome some of the difficulties of rare disease research where geographical limitations and small population sizes (ergo small sample sizes) lead to statistical power issues. In EMA, within-person analyses are possible, even with small sample sizes, due to the high frequency of assessments. It also allows for highly specific measurements of individuals [26] which for a broad-spectrum disease (like TSC), can be invaluable for understanding each individual presentation.

To be effective, Smart EMA should follow the recommendations described by the participants in the current study. The EMA app should be simple, quick and not complicated to use, and participants should be sufficiently trained. The app should be fun to use, or participants should receive rewards or incentives to motivate them. Although participants described how they would like to know when the EMA questions would come, this would contradict the increased ecological validity and reduce reactivity that a random or semi-random EMA provides. To balance this request, participants should be given a background of EMA (designed to their intellectual ability) and given reasons why the EMA questionnaires may be random and that it is expected that not all the questionnaires will be answered due to this reason. This may alleviate some anxiety for participants who are trying to balance their commitment to the research and to their daily responsibilities (i.e. work).

### Limitations

Even though we compared our sample to the best estimate UK-representative sample (TS 2000 study) and found no statistical differences (age, gender, age at diagnosis and TSC genotype), the representativeness of our sample is limited. Due to ethical limitations, only young adults who could consent for themselves were eligible for the study. This will have excluded some young adults with TSC who require legal guardianship. Furthermore, no participants reported an ID, which is common in the TSC population, and often coincides with less use of the internet or smartphones [75]. This digital divide may prevent Smart EMA from being feasible for all young adults with TSC. However, Bakkum et al. [69] interviewed participants with mild-moderate ID about the acceptability of EMA methods and found similar facilitators to our participants including the need for fixed timing and adaption around life constraints. This suggests EMA could be adapted for individuals with ID. Within the current study, focus groups with parents of young adults with TSC were also run and many of their young adults were reported to have ID. Due to the differences between the two samples and the data gathered, the results of this have been reported in a separate article. Despite there being no adults with ID in our study, the adults who are more mildly affected are still likely to benefit from Smart EMA as they can still suffer from TAND issues. Their transition experiences are also difficult, and it is important to understand support and independent living for those without ID. Following these two studies, a further extension will be to include young adults with TSC who lack capacity and may not use smartphones, to explore ways in which Smart EMA may be beneficial to them and their families.

Even though examples of EMA apps were shown, some of the participants did struggle with the hypothetical concept of Smart EMA and therefore were limited in their descriptions of potential barriers and facilitators of using Smart EMA. Some often commented that they would have more of an idea “when they used the app”. If possible, researchers should try and provide test versions of different EMA apps to allow participants to experience the app first-hand to limit the number of hypothetical concepts.

Another limitation was that two modes of qualitative data collection were utilised in this study, focus groups and interviews, which can serve different purposes and produce different data. Focus groups were chosen as the main methodology as they can be used in exploratory areas of healthcare research [76, 77] and facilitate discussion, especially in young adults, as they feel supported by their peers, which balances the power between themselves and the researcher [78]. However, due to the prevalence of TAND symptoms, accessibility of this study was of high importance and key information would have been lost from the five participants who chose to be interviewed if this option was not available.

Even with the addition of interviews, our sample size is still small and does not reach the reported ‘number’ for data saturation [79, 80]. Time resources and a limited population to recruit from impacted our sample size. However, after multiple iterations of the framework between the two coders, it was felt that the codebook captured our interpretation of all the meaningful codes. However, we do acknowledge that meaning resides in our interactions as researchers with the data and that there may always be new interpretations from the data, depending on the interpreter [81]. Due to this, we prioritise the meaningfulness of our codes and themes in answering our research question and let the participant respondent validation guide our confidence in the results.

### Future Smart EMA feasibility research requirements

The parameters of a Smart EMA study can vary greatly however, methodological evaluation of Smart EMA specifications is lacking, especially in individuals with health conditions. Patient and Public Involvement (PPI) is crucial to guide the design in the absence of methodological best practice. One example is Dewa et al. [82], who successfully co-produced a Smart EMA study with young adults who had experienced psychiatric inpatient care. This study has highlighted important aspects of Smart EMA that need to be addressed for a young adult TSC population. Therefore, the next stage of the study will be to integrate these findings alongside co-developing an EMA protocol with young adults with TSC using an advisory group. The app will then be tested with a larger group of young adults with TSC and assessed for feasibility and acceptability via compliance rates and post-EMA interviews.

## Conclusion

This qualitative study investigated the attitudes and opinions of young adults with TSC on the accessibility and appropriateness of using Smart EMA in their daily lives. Several themes around how the young adults would use Smart EMA emerged, through helping manage the transition from childhood to adulthood, to helping understand and communicate their emotions and feelings. Hypotheses for the moderation of Smart EMA were generated: Unpredictability and burden, accessibility and personalisation, familiarity and ease and rare disease motivation may all influence engagement with Smart EMA. These variables should be evaluated in future studies to contribute to the feasibility and acceptability of Smart EMA with young adults with TSC and other rare genetic diseases.

## Electronic supplementary material

Below is the link to the electronic supplementary material.

Supplementary Material 1

## Data Availability

The datasets generated and/or analysed during the current study are not publicly available due to information that could compromise research participant privacy/consent but are available from the corresponding author on reasonable request.

## Abbreviations

EMA: Ecological Momentary Assessment
FG: Focus Group
ID: Intellectual Disability
INT: Interview
KSP: Key Support Person
LTC’s: Long Term Conditions
NF1: Neurofibromatosis type 1
PPI: Public Patient Involvement
SD: Sandhoff Disease
Smart EMA: Smartphone EMA
TAND: TSC-Associated Neuropsychiatric Disorders
TSA: Tuberous Sclerosis Association
TSC: Tuberous Sclerosis Complex
TSD: Tay-Sachs Disease
TS 2000 Study: Tuberous Sclerosis 2000 Study
TYP: TSC Young Person
