# Supplementary material for: Exploring the use of smartphone monitoring for young adults with Tuberous Sclerosis Complex (TSC): a qualitative analysis
Source: Orphanet J Rare Dis. 2026 Apr 22;21:165. doi: 10.1186/s13023-026-04287-5 (PMC13101228; doi:10.1186/s13023-026-04287-5)
Supplement: Supplementary file 1 — Supplementary Material 1 [file 13023_2026_4287_MOESM1_ESM.docx]

**ADDITIONAL FILES**

**Additional File 1**

**Reflexivity Statement**


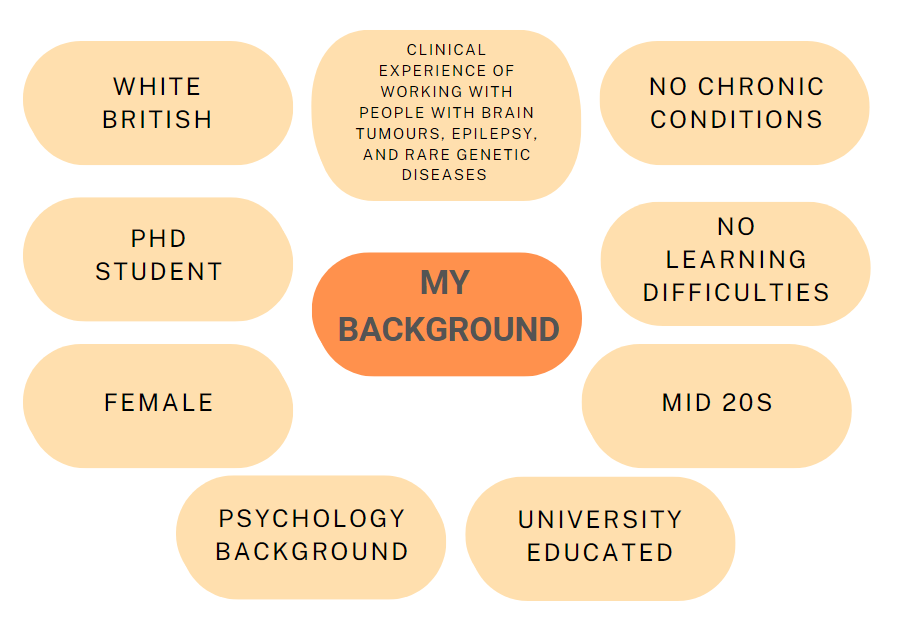


When designing the study, I was most aware of my position as a university-educated individual with a psychology background and without personal experience of a chronic condition or intellectual difficulties. I was also concerned about my knowledge of the methodology of ‘EMA’ as I had decided to simplify the information given to the participants and only explain it as an ‘app’ rather than explain in detail ‘EMA’ methodology. Although I provided some background information on the ‘app’ and the focus group and interview schedule were designed to be answered with the information provided, I was wary that my knowledge of EMA may influence any of my spontaneous follow-up prompts and therefore be difficult to answer.

From the start of data collection, I quickly began to observe the wide spectrum of abilities of young people with TSC. Because of my professional background and how much the participants needed to rely on their parents, I noticed that some individuals struggled in their intellectual abilities. Some seemed intellectually able though. Because of this continuum, I was worried about the nature of the focus group and whether some participants would be able to access and participate successfully in the groups. I was less worried about the interviews as I believed I could adapt my interview style to match the participant’s ability.

With some participants, especially those who seemed to have intellectual difficulties, I was surprised by how much they engaged with the interview. This was probably due to my bias that those with intellectual difficulties do not say much however most were very willing to give their opinion. In the first interview, I felt my questioning could have been a bit more open however I thought the participant might not be able to understand open questions so rather I asked a closed simple one first and then asked why. This led to me realising that because there were two aims to this study, I sometimes felt that they conflicted. For example, when interviewing patients with intellectual difficulties, I gave more examples to the questions to support their understanding. For example, “Would you change anything about the structure?” If they demonstrated confusion, I would provide examples such as “so for example, the number of questions, time to complete the questions, how many days you use the app…” This was helpful for the aim of designing the next study, but it reduced how qualitatively useful their response was as often only used my examples and not their ‘original ideas’.

I think the combination of individuals' intellectual abilities and the young people’s shyness led to the focus groups being quite stilted. I as the interviewer had to speak and ask more direct questions than I wanted to. It also led to little back and forth between participants in the groups and often required me to prompt “Does anyone else feel the same or different?”. One result of this was that it did make it easier to combine the data of the focus groups and interviews as they felt similar in their presentation. In the focus groups, there were instances of participants seeking ideas and support from the other participants which sanctioned my choice of using focus groups as the primary option. But, as a few participants requested an interview instead, I was glad I was able to give them an opportunity to share their experiences and contribute to the research. However, I was still aware of the difference between the data gathered by the two modes.

One difference I found between the two methods was that it was harder to not go into ‘therapy mode” in the interview compared to the focus groups. My background experience of working clinically with similar individuals (rare disease, epilepsy, intellectual disability, brain tumours etc) hindered my ability to stay as a neutral researcher. I often found myself trying to repeat back their answers to show my understanding and acknowledge their difficulties in a therapeutic way rather than a researcher. I did adapt as the interviews went on as I learnt to navigate those conversations without feeling the need to provide ‘supportive feedback’ and instead, prioritise prompting further description of their experiences and feelings. On reflection, my clinical skills may have helped with rapport building which in turn could have allowed the participants to share more. As such, I believe there are unique benefits to being a clinical researcher.

In summary, at first, I identified myself as an “outside researcher” (Hellawell, 2007) as I had no personal experience with living with a rare disease or having intellectual difficulties. However, as the focus groups and interviews went on, I realised my background experience of working with children and young adults with very similar conditions had changed both the way I ran the focus groups and interviews and my assumptions and biases about the participants. I acknowledged that this would influence my interpretation of their experiences.

Hellawell, D. (2007). Teaching in Higher Education Inside-out: analysis of the insider-outsider concept as a heuristic device to develop reflexivity in students doing qualitative research. https://doi.org/10.1080/13562510600874292

**Additional File 2**

**Technology Use Questionnaire**

1. Do you currently use a mobile phone?
   1. Yes
   2. No
2. Do you currently use a 'smartphone' (A phone that can access the internet)?
   1. Yes
   2. No
3. How long have you owned a smartphone?
   1. Years ______
   2. Less than a year
4. For which of the following activities do you ever use your smartphone for? (Check all that apply)
   1. Phone calls
   2. Text messaging
   3. Shopping
   4. Banking
   5. Emailing
   6. Exercising
   7. Social networking (e.g., Facebook)
   8. Navigating with maps (e.g., finding a store)
   9. Entertainment (e.g., movies, games)
   10. Other, please state
5. Do you have any 'apps' that help track or manage your health? (For example My Fitness Pal or Headspace)
   1. Yes, please state __________
   2. No
6. Do you currently use any other electronic device that can access the internet such as a tablet, laptop or computer?
   1. No
   2. Yes, please state __________
7. On these devices do you have any 'apps' that help track or manage your health
   1. No
   2. Yes, please state __________
8. How easy is it for you to use technology that accesses the internet such as smartphones, tablets, computers etc?
   1. Extremely difficult
   2. Somewhat difficult
   3. Neither easy nor difficult
   4. Somewhat easy
   5. Extremely easy
9. Do you use any wearable technology (for example a smartwatch or a fitness tracker such as an Apple Watch, Samsung Galaxy, Fitbit)?
   1. No
   2. Yes, please state __________

**Additional File 3**

**Word Clouds**

**Focus Group 1**


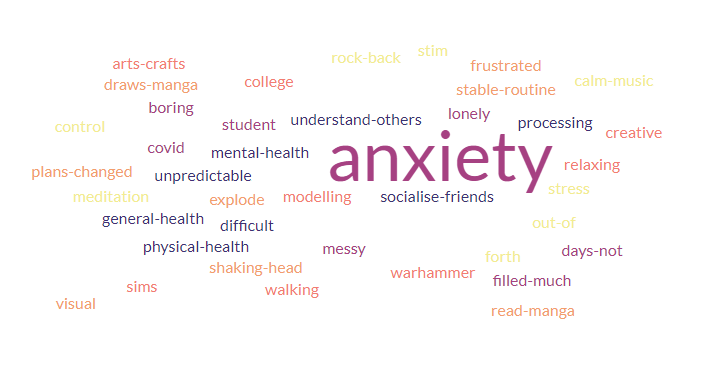


**Focus Group 2**


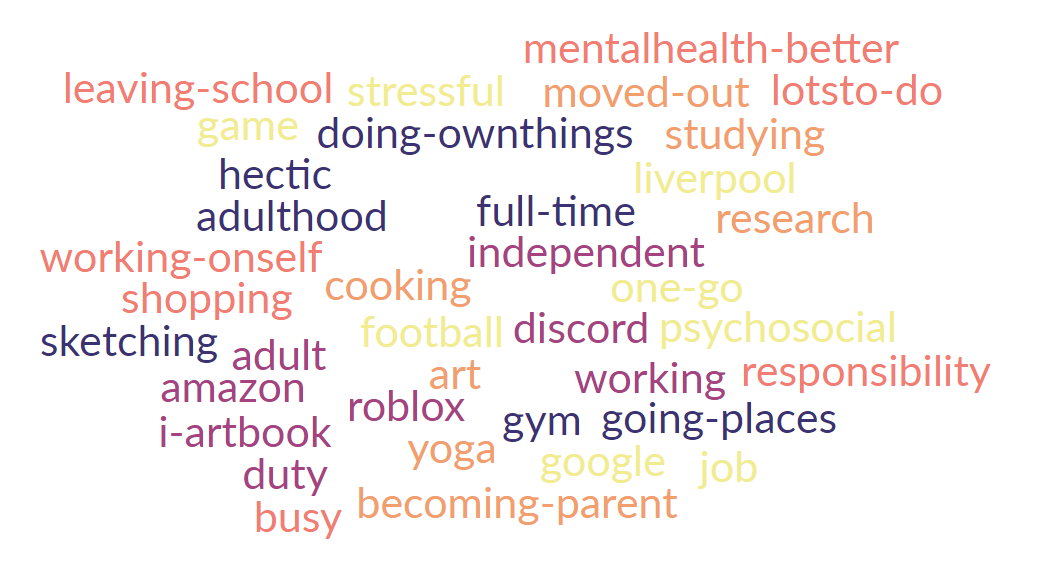


**Interview 1**


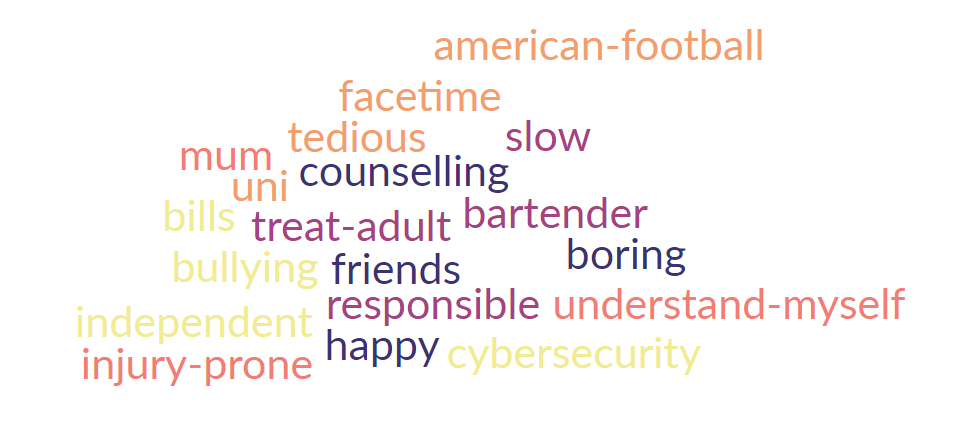


**Interview 2**


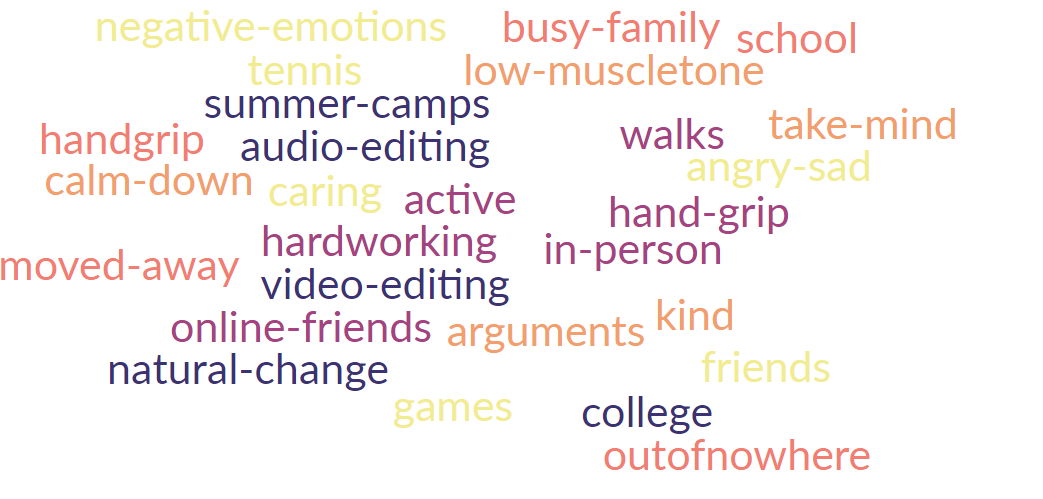


**Interview 3**


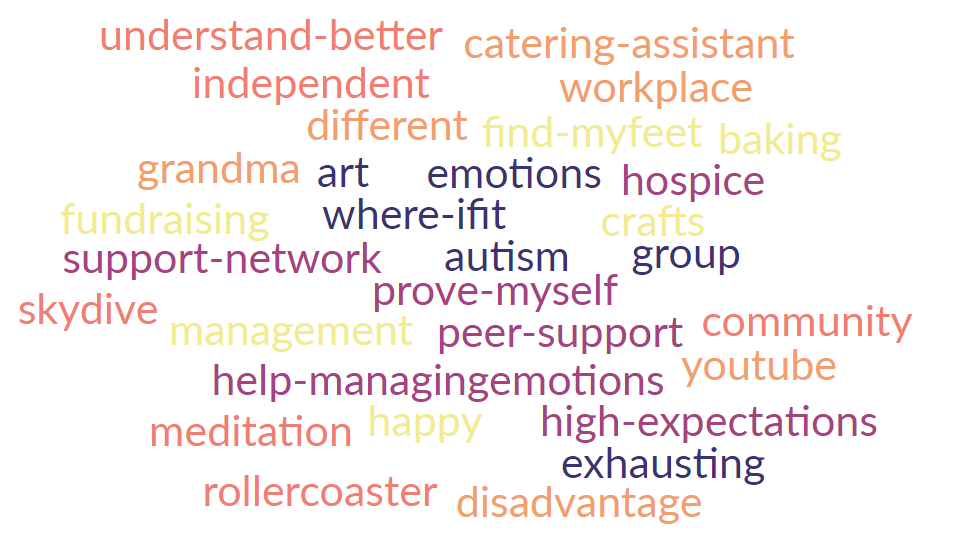


**Interview 4**

**
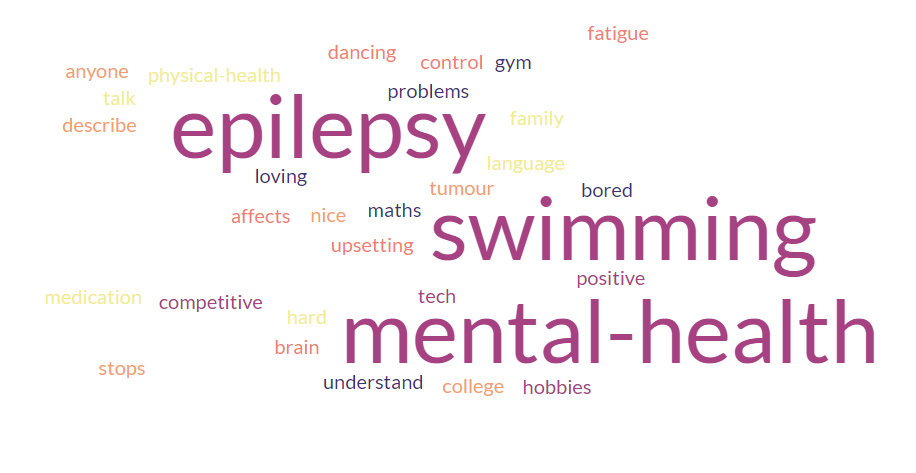
**

**Interview 5**

**
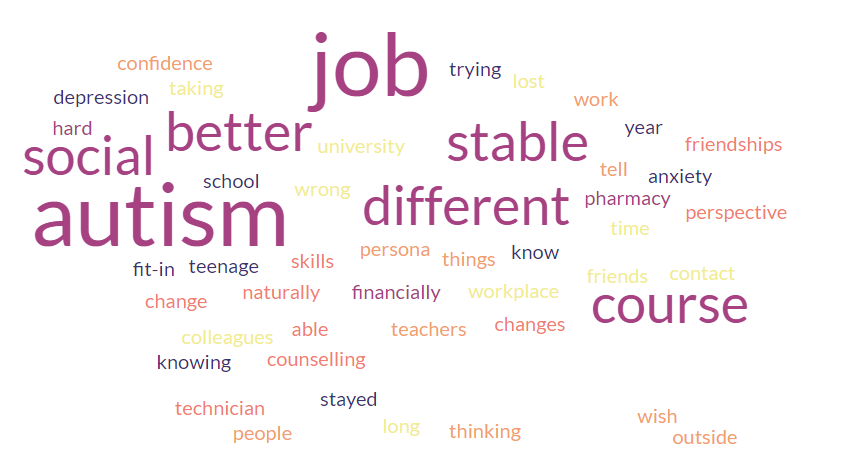
**

**Additional File 4
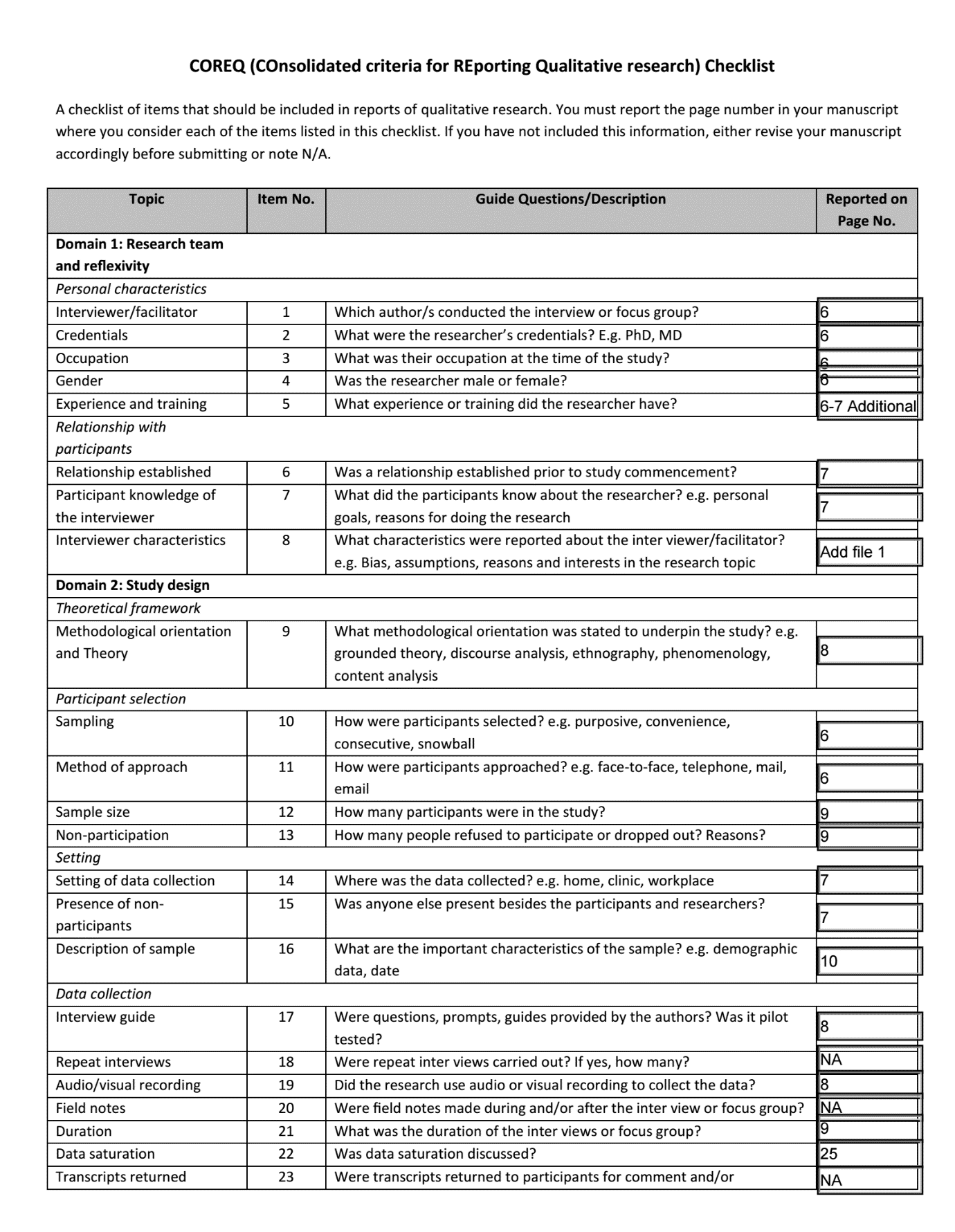
**

**
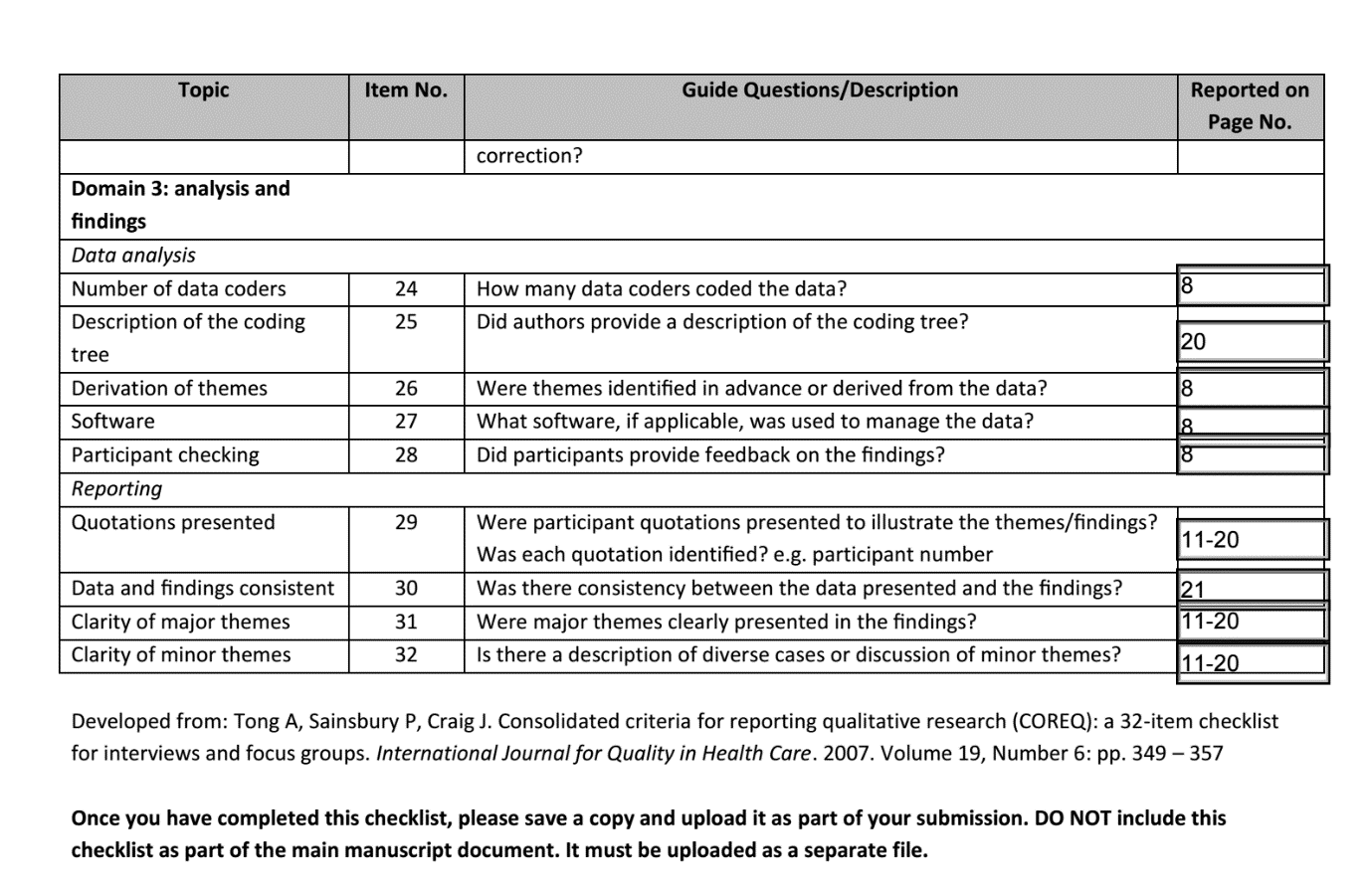
**
